# Inhibition of Enterovirus A71 by a Novel 2-Phenyl-Benzimidazole Derivative

**DOI:** 10.3390/v13010058

**Published:** 2021-01-04

**Authors:** Roberta Ibba, Antonio Carta, Silvia Madeddu, Paola Caria, Gabriele Serreli, Sandra Piras, Simona Sestito, Roberta Loddo, Giuseppina Sanna

**Affiliations:** 1 Department of Chemistry and Pharmacy, University of Sassari, Via Muroni, 23A, 07100 Sassari, Italy; ribba@uniss.it (R.I.); piras@uniss.it (S.P.); ssestito@uniss.it (S.S.); 2 Department of Biomedical Sciences, University of Cagliari, Cittadella Universitaria Monserrato (Cagliari), 09042 Monserrato, Italy; silvia.madeddu@unica.it (S.M.); paola.caria@unica.it (P.C.); gabriele.serreli@unica.it (G.S.); rloddo@unica.it (R.L.)

**Keywords:** EV-A71, neurological complications, antivirals, benzimidazole derivatives, time course, penetration assay, apoptosis assay, TEER

## Abstract

Enterovirus A71 (EV-A71) infection has emerged as a significant public health concern at the global level. Epidemic events of EV-A71 have been reported worldwide, and this succession of outbreaks has heightened concern that EV-A71 may become a public health threat. In recent years, widespread A71 enterovirus also occurred in European countries. EV-A71 infection causes hand-foot-mouth disease (HFMD), herpangina, and fever. However, it can sometimes induce a variety of neurological complications, including encephalitis, aseptic meningitis, pulmonary edema, and acute flaccid paralysis. We identified new benzimidazole derivatives and described theirin vitrocytotoxicity and broad-spectrum anti-enterovirus activity. Among them, derivative **2b** resulted in interesting activity against EV-A71, and therefore it was selected for further investigations. Compound **2b** proved to be able to protect cell monolayers from EV-A71-induced cytopathogenicity, with an EC_50_ of 3 µM. Moreover, Vero-76 cells resulted in being significantly protected from necrosis and apoptosis when treated with **2b** at 20 and 80 µM. Compound **2b** reduced viral adsorption to Vero-76 cells, and when evaluated in a time-of-addition assay, the derivative had the highest effect when added during the infection period. Moreover, derivative **2b** reduced viral penetration into host cells. Besides, **2b** did not affect intestinal monolayers permeability, showing no toxic effects. A detailed insight into the efficacy of compound **2b** against EV-A71 showed a dose-dependent reduction in the viral titer, also at low concentrations. Mechanism of action investigations suggested that our derivative can inhibit viral endocytosis by reducing viral attachment to and penetration into host cells. Pharmacokinetic and toxicity predictions validated compound **2b** as a good candidate for furtherin vivoassays.

## 1. Introduction

Enterovirus A-71 (EV-A71) is a positive-strand RNA virus belonging to the *Picornaviridae* family, genus *Enterovirus*, commonly associated with mild diseases in children. EV-A71 clinical manifestations include hand-foot-mouth disease (HFMD), herpangina, and fever, but it also may involve the central nervous system (CNS). Neurological manifestations comprise meningitis, brainstem encephalitis, and acute flaccid paralysis. Epidemic events of EV-A71 have been reported in the last ten years in Australia, Japan, Malaysia, Taiwan, Vietnam, and China, and this succession of outbreaks has heightened concern that EV-A71 may become a public health threat [1,2]. In recent years, widespread A71 infections occurred also in European countries, such as the Netherlands, France, and Spain (https://www.eurosurveillance.org). HFMD occurrences constitute a perpetual menace in China, resulting in 7.2 million cases between 2008 and 2012 [3]. EV-A71 emerged as responsible for about 80% of occurred severe cases and 93% of the deaths during this time [4]. As a major neurotropic causative agent of HFMD, EV-A71 has replaced poliovirus as the most clinically important enterovirus following the global eradication of poliomyelitis [5,6]. HFMD is a benign and common childhood disease, easily transmitted via the fecal–oral routes and characterized by mucocutaneous ulcerative vesicles in the mouth as well as lesions on the hands and feet. While HFMD is caused by several members of the Enterovirus genus, HFMD caused by EV-A71 is considered to be the most pathogenic. Fatal neurological complications have been reported in children, with clinical symptoms such as aseptic meningitis, poliomyelitis-like acute flaccid paralysis, and brainstem encephalitis that may lead to cardiovascular collapse and pulmonary edema [7]. Seven EV A71 genogroups are known (A–G) [8]. Genogroup A contains the prototype strain BrCr, which was isolated in the United States from a 2-month-old male with aseptic meningitis, but it was also documented in China in 2008. Viruses belonging to genogroups B and C have been circulating more largely and contain six and five genotypes (B0–B5 and C1–C5). Genogroups D and G have been documented in India and genogroups E and F in Africa were recognized more recently. Several countries within the Asia-Pacific region have reported large outbreaks of EV-71 related to new genotypes B and C. Most of the EV-A71 strains circulating in Europe belong to genotype C [8].

Up until now, two inactivated vaccines have been developed and marketed exclusively in China [9]. With no established antiviral treatment for EV-A71 infection [10], management of severe EV-A71 infections is mainly aimed at alleviating disease symptoms using supportive treatments such as mechanical cardiopulmonary support systems and the administration of milrinone to prevent cardiopulmonary failure and improve clinical outcome in patients [11]. Other treatments include intravenous administration of immunoglobulin, interferon-alpha, and corticosteroids, all leading to favorable clinical outcomes in patients. With currently limited antivirals against EV-A71, it is fundamental to develop techniques and strategies to expand the drug discovery pipeline [12]. 

Among a long-lasting antiviral discovery project, we synthesized several small molecules that were subjected to a wide antiviral [13,14,15] and anti-infective screening [16,17]. Some of them turned out as active against representative Enteroviruses [18]. Previously reported benzotriazole derivatives which showed antiviral activity against Coxsackievirus B5 [18] and respiratory syncytial virus (RSV) [19] were used as prototypes for a scaffold simplification strategy and bioisostere substitutions. By the scaffold simplification strategy, the side chain on the main scaffold was reduced in size, choosing small substituted phenyl moieties. The benzotriazole scaffold was replaced by a bioisostere benzimidazole skeleton. They were selected as lead compounds and underwent classical chemical scaffold modification in order to improve potency and selectivity. In this study, we focused on identifying promising enteroviruses drug candidates and anew series of variously substituted 2-phenyl-benzimidazole derivatives (**1a,b-5a,b**) was designed, synthesized, and tested against a panel of relevant enteroviruses.

## 2. Materials and Methods

### 2.1. Chemistry

#### 2.1.1. Synthesis

All starting materials were purchased from Sigma-Aldrich (Merck KGaA, Darmstadt, Germany). Benzimidazole ring closure, to obtain derivatives **1a**,**b**-**5a**,**b,** was carried out as described in the literature [20] by mixing *o*-phenylenediamines (**1**-**5**) and commercial aldehydes (**a** and **b**) in a ratio of 1:1,dissolvingthe mixture in a few mL of acetonitrile (CH_3_CN), and adding H_2_O_2_ 30% (ratio 1:7) and HCl 37% (ratio 1:3.5) at room temperature for a proper time, generally a few hours to a maximum of an overnight time period. The solid product obtained was filtered off, washed with CH_3_CN and then with water until neutral pH, and ultimately dried overnight in an oven. Products were obtained as pure solids by crystallization from ethanol and then characterized. 

#### 2.1.2. Chemical Characterization

Compounds’ melting points (m.p.) were taken in open capillaries in a Köfler hot stage and are uncorrected. Retention factors (R_f_) were measured by thin-layer chromatography (TLC) using Merck F-254 commercial plates and a proper mixture of petroleum ether (PE) and ethyl acetate (EA) as eluent. Nuclear magnetic resonance (NMR) spectra were registered in solutions in deuterated DMSO or acetone and were recorded with a Bruker Avance III 400 NanoBay (400 MHz) instrument. ^1^H-NMR chemical shifts are reported in parts per million (ppm) downfield from tetramethylsilane (TMS) used as internal standard, at 400 MHz. Chemical shift values are reported in ppm (*δ*) and coupling constants (*J*) are reported in Hertz (Hz). Signal multiplicities are represented as s (singlet), d (doublet), dd (doublet of doublets), ddd (doublet of doublet of doublets), t (triplet), q (quadruplet), and m (multiplet). ^13^C-NMR chemical shifts are reported downfield from tetramethylsilane (TMS) used as internal standard, at 100 MHz.A suitable method among the APT (attached proton test) and jmod (J-modulated spin-echo for X-nuclei coupled to H-1 to determine the number of attached protons) was selected for each compound. The solutions for ESI-MS measurements were prepared by dissolving the solid compounds in HPLC acetonitrile to obtain a concentration of 1.0–2.0 ppm. Mass spectra in the positive-ion mode were obtained on a Q Exactive Plus Hybrid Quadrupole-Orbitrap (Thermo Fisher Scientific, Waltham, MA, USA) mass spectrometer. The solutions were infused at a flow rate of 5.00 μL/min into the ESI chamber. The spectra were recorded in the *m/z* range 150–800 at a resolution of 140,000 and accumulated for at least 2 min in order to increase the signal-to-noise ratio. The instrumental conditions used for the measurements were as follows: spray voltage 2300 V, capillary temperature 250 °C, sheath gas 10 (arbitrary units), auxiliary gas 3 (arbitrary units), sweep gas 0 (arbitrary units), and probe heater temperature 50 °C. ESI-MS spectra were analyzed by using Thermo Xcalibur 3.0.63 software (Thermo Fisher Scientific), and the average deconvoluted monoisotopic masses were obtained through the Xtract tool integrated within the software. 

### 2.2. Biology

#### 2.2.1. Cells and Viruses

Cell lines were purchased from the American Type Culture Collection (ATCC). The absence of mycoplasma contamination was checked periodically by the Hoechst staining method. Cell lines supporting the multiplication of Enteroviruses were the following: Monkey kidney (Vero-76) [ATCC CRL 1587 Cercopithecus Aethiops], Human cervix adenocarcinoma (HeLa cells) [ATCC CCL-2], and Macaca mulatta, monkey, rhesus (LLC-MK2) [ATCC CCL-7]. Caco-2 cells (ECACC Salisbury, Wiltshire, UK) were cultured in Dulbecco’s modified Eagle’s medium (DMEM), supplemented with 10% heat-inactivated bovine serum, 2 mM l-glutamine, 1% non-essential amino acids, 100 U/mL penicillin, and 100 mg/mL streptomycin, in monolayers at 37 °C in a humidified atmosphere of 5% CO_2_, [21] replacing the medium twice a week. For experimental studies, Caco-2 cells, at passage 21–40, were plated and used 18–21 dayspost-seeding, as differentiated enterocytes. Viruses were purchased from the American Type Culture Collection (ATCC). Viruses representative of positive-sense, single-stranded RNAs (ssRNA+) were: Picornaviridae: human enterovirus B [coxsackie type B4 (CV-B4), strain J.V.B. (ATCC VR-184), coxsackie type B5 (CV-B5), strain Faulkner (ATCC VR-185), coxsackie type B3 (CV-B3), strain Nancy (ATCC VR-30)], human enterovirus B [echovirus 9], human enterovirus A71 strain BrCr (ATCC VR-1775),enterovirus C [poliovirus type-1 (PV-1), Sabin strain (Sb-1) Chat (ATCC VR-1562)],and human enterovirus D 68 strain Fermon (ATCC VR-1826)]. 

#### 2.2.2. Cytotoxicity Assays

HeLa and LLC-MK2 cells were seeded in 96-well plates at an initial density of 5 × 10^5^ cells/mL, in Minimum Essential Medium with Earle’s salts (MEM-E), l-glutamine, 1 mM sodium pyruvate, and 25 mg/L kanamycin, supplemented with 10% fetal bovine serum (FBS). Vero-76 cells were seeded in 96-well plates at an initial density of 5 × 10^5^ cells/mL, in Dulbecco’s modified Eagle medium (D-MEM), with l-glutamine and 25 mg/L kanamycin, supplemented with 10% FBS. Cell cultures were then incubated at 37 °C in a humidified, 5% CO_2_ atmosphere, in the absence or presence of serial dilutions of test compounds. The test medium used for the cytotoxic assay as well as for the antiviral assay contained 1% of the appropriate serum. Cell viability was determined after 72–96 h at 37 °C by the MTT method for HeLa and Vero-76 as previously reported [22]. 

#### 2.2.3. Antiviral Assays

Compounds’ activity against EV-A71 and EV-D68 was based on inhibition of virus-induced cytopathogenicity in Vero-76 and HeLa cells, respectively, acutely infected with an m.o.i. of 0.01. Briefly, Vero-76 and HeLa cells were seeded in 96-well plates at a density of 2 × 10^4^ and 3 × 10^4^ cells/well, respectively, and were allowed to form confluent monolayers by incubating overnight in growth medium at 37 °C in a humidified CO_2_ (5%) atmosphere. Cell monolayers were then infected with 200 and 300 PFU (50 μL of a proper virus dilution) in maintenance medium (D-MEM and MEM-Earl, with l-glutamine, 1 mM sodium pyruvate, and 0.025 g/L kanamycin, supplemented with 0.5% inactivated FBS, respectively) to give an m.o.i of 0.01. After 2 h, 50 μL of maintenance medium, without or with serial dilutions of test samples, wasadded. After a 3/4-day incubation at 37 °C, cell viability was determined by the MTT method [23]. Compounds’ activity against CV-B3, CV-B4, CV-B5, and Echo 9 was determined by plaque reduction assays in infected cell monolayers, as previously reported [24]. Plc (Pleconaril), NM107 (2′-C-methylcytidine),and Rup (Rupintrivir) were used as references and internal controls.

#### 2.2.4. Yield Reduction Assay

Vero-76 cells were inoculated with EV-A71 at an m.o.i. of 0.1 in maintenance medium and tested compounds at non-cytotoxic concentrations. Following the 2-h adsorption period at 37 °C and 5% CO_2_, the inoculum was removed and replaced with fresh medium containing the same concentration of compounds. After 96 h at 37 °C and 5% CO_2_, each sample was harvested and diluted with serial passages, starting from 10^−1^ up to 10^−8^. The titer of the serial dilutions of the virus-containing supernatant was determined by a standard plaque assay, counting the number of obtained plaques in at least two different dilutions for each concentration. NM107 (2′-C-Methyl-Cytidine) was used as a reference compound.

#### 2.2.5. Apoptosis Assay

To assess levels of apoptosis following **2b** derivative treatment, a flow cytometric analysis, using the cell apoptosis kit Annexin V/Propidium Iodide (PI) double staining uptake (Invitrogen, Life Technologies, Italy), was used. Vero-76 cells, at the density of 2 × 10^5^ cells/mL, were seeded in 12-well plates (Corning, New York, NY, USA) with complete medium (described in cell culture section). After EV-A71 viral adsorption, the cells were incubated in the absence or presence of different concentrations of **2b** for 96 h, until the cytopatic effect CPE of the virus control reached 70–80%. Cells were then washed once with PBS 1 X and re-suspended in 100 μL of Annexin binding buffer plus 1 μL of Annexin V and 1 μL of PI. Then, the reaction was performed in the dark for 15 minutes at room temperature. Stained cells were then analyzed by flow cytometry, measuring the fluorescence emission at 530 and 620 nm using a 488 nm excitation laser (MoFloAstrios EQ, Beckman Coulter, Pasadena, CA, USA). Cell apoptosis was analyzed using the software Summit Version 6.3.1.1, Beckman Coulter. 

#### 2.2.6. Virucidal Activity Assay

Compound **2b** (20 µM) was incubated with 1 × 10^5^ PFU/mL of EV-A71 at either 4 or 37 °C for 1 h. The mixture without a test sample was used as the control. At the end of the incubation period, samples were serially diluted in media, and titers were determined on Vero-76 cells at high dilutions, at which the compound was not active. Virus titers were determined by a plaque assay in Vero-76 cells.

#### 2.2.7. Cell Pretreatment Assay

Vero-76 cell monolayers in 24-well plates were incubated with 20 µM concentration of the **2b** or 50 µM of NM 107 for 2 h at 4 °C. After the removal of the compounds and two gentle washes, cells were infected with EV-A71. After virus adsorption to cells, the inoculum was removed and the cells were then overlaid with medium, incubated for 4 days at 37 °C, and then virus titers were determined by a plaque assay.

#### 2.2.8. Adsorption Assays

Vero-76 cells grown in 24-well plates were infected with EV-A71, with an m.o.i. of 1, in the presence or absence of compound **2b**. Multiwells were incubated for 120 min at 4 °C. The medium containing the unabsorbed virus was then removed, and cells were washed twice with PBS and overlayed with medium. Plaques were counted after 96 h of incubation at 37 °C.

#### 2.2.9. Time-of-Addition Assay

The confluent monolayers of Vero-76 cells in 24-well tissue culture plates were infected for 1 h at room temperature with EV-A71 dilutions to give a final m.o.i. of 1. After adsorption, the monolayers were washed twice with maintenance medium and incubated with the same medium at 5% CO_2_ and 37 °C (time zero). Vero-76 cells were treated with compound **2b** (20 μM, approximately 10 times higher than the EC_50_) or the reference for 1 h during the infection period (at −1 to 0) and at a specific time point, 0 to 2, 2 to 4, 4 to 6, 6 to 8, and 8 to 10 h post-infection. After each incubation period, the monolayers were washed two times with maintenance medium and incubated with fresh medium until 12 h post-infection. Then, the monolayers were frozen at -80 °C and the viral titers were determined by a plaque assay.

#### 2.2.10. Penetration Assay

A 24-well tissue culture plate was seeded with Vero-76 cells (4 × 10^5^ cells/well), which were then incubated overnight at 37 °C and 5% CO_2_. The cells were chilled on ice for 1 h, and the medium was removed. The cells were infected with 100 PFU (200 µL) of EV-A71 on ice for 120 min. The medium containing the unbound virus was then removed; various concentrations of compound **2b** (100–0.8 µM) in medium were added, and the cells were incubated at 37 °C for 60 min to trigger endocytosis of the virus. The infected cells were then treated with alkaline phosphate-buffered saline (PBS; pH 11) for 1 min to inactivate any viruses that had not penetrated the cells, and then acidic PBS (pH 3) was immediately added to neutralize the mix. The neutralized medium was removed, and cells were overlayed with 0.75% methylcellulose in media and then incubated at 37 °C. After incubation at 37 °C for 96 h, cells were stained, and plaques were determined by counting. This penetration assay was performed according to the method [25,26].

#### 2.2.11. Evaluation of Cell Monolayer Permeabilization (TEER)

The activity of compound **2b** on intestinal cells monolayer permeability was assessed by measuring the transepithelial electrical resistance (TEER) value. Caco-2 cells (0.5 × 10^5^ cells/well) were grown in 12 mm i.d. Transwell inserts (polycarbonate membrane, 0.4 μm pore size) and culture medium was dispensed in the apical (0.5 mL) and basolateral (1.5 mL) compartments of each well. Resistance was measured using a Millicell–ERS ohmmeter (Millicell-ERS system, Millipore, Bedford, MA, USA) as previously reported by Serreli et al., [27]. Once the cell monolayers were formed, only inserts with TEER values >300 Ω/cm^2^ were chosen for the experiment. Then, compound **2b** (final concentration 20 µM) and, to induce an increase in permeability, an oxysterol mixture (final concentration 60 µM) prepared as described by Serra et al. [28] were added to the culture medium. TEER values were subsequently measured at intervals of 0.25, 0.5, 0.75, 1, 2, 3, 6, 18, 24, and 48 h and are reported as a percentage of the corresponding TEER value at time zero (T = 0).

#### 2.2.12. Statistical Analysis

Cell-based experiments were independently repeated at least three times. The data are reported as mean ± standard deviation (SD). The statistical significance values are defined as * *p*< 0.05, ** *p*< 0.01, *** *p*< 0.001. The statistical significance was calculated with the Mann–Whitney test performed in GraphPad Prism (San Diego, CA, USA).

#### 2.2.13. Linear Regression Analysis

The extent of cell growth/viability and viral multiplication, at each drug concentration tested, were expressed as percentage of untreated controls. Concentrations resulting in 50% inhibition (CC_50_ or EC_50_) were determined by linear regression analysis.

### 2.3. In Silico Screening

The PreADMET (http://preadmet.bmdrc.kr/) tool was used to calculate druglikeness for the library of new compounds (**1a**-**5a**, **1b**-**5b**). An ADME (absorption, distribution, metabolism and excretion) profile for all the new derivatives was predicted using the PreADMET and SwissADME (http://www.swissadme.ch/) tools. Two-dimensional structural models for each compound were drawn in ChemDraw Ultra version 12.0 (Cambridge Software) and SMILES (Simplified Molecular Input Line Entry System) strings of each compound were used as input for the predictions. Degree of plasma protein binding (PPB) was calculated with PreADMET and is classified as strongly bound if PPB% > 90% and weakly bound if PPB% < 90%. Blood–brain barrier (BBB) penetration was predicted by both the tools. BBB penetration is presented as a ratio of compound concentration in brain (C_brain_) and compound concentration in blood (C_blood_). Compounds are labeled as high, middle, or highly absorbing into the CNS when C_brain_/C_blood_ values are >2, 2–0.1, and <0.1, respectively. SwissADMET evaluates whether a compound would be absorbed into the CNS or not. Lipophilicity was calculated by SwissADME and reported as Consensus Log P_o/w_ (CLog P_o/w_) as an average of different predictions from five different algorithms (iLOGP, XLOGP3, WLOGP, MLOGP, SILICOS-IT). Water solubility was evaluated by the SwissADME tool. It also evaluated compounds as targets of P-glycoprotein (P-gp) efflux. Gastrointestinal (GI) absorption was predicted by SwissADME, while PreADMET used Caco-2 cells as a GI absorption model and permeation was measured as nm/s. Permeation was classified as low, middle, or high for values <4 nm/s, 4–70 nm/s, and >70 nm/s, respectively. PreADMET predicts the toxicity of compounds on models of the Ames test against strains of *Salmonella typhimurium* TA100 and TA1535. Toxicity was calculated with or without consideration of liver metabolism as metabolic activation by a rat liver homogenate (S9 fraction). Results were produced as positive or negative mutagenicity. 

## 3. Experimental Section

### 3.1. 2-(4-Isopropylphenyl)-1H-Benzo[d]imidazole (**1a**)

Compound **1a** (C_16_H_16_N_2_, MW 236.312) was obtained in a total yield of 88%; m.p. 206–207 °C; TLC (PE/EA 7/3): R_f_ 0.62. ^1^H-NMR (DMSO-d_6_): δ 8.11 (2H, d,J = 8.6 Hz, H-2′,6′), 7.66 (1H, d, J = 7 Hz, H-4), 7.61 (2H, d, J = 8.6 Hz, H-3′,5′), 7.54 (1H, d, J = 7 Hz, H-7), 7.21 (2H, m, H-5,6), 1.34 (9H, s, 3CH_3_). ^13^C-NMR (jmod, DMSO-d_6_): δ 152.49 (C), 151.18 (C), 142.98 (C), 136.94 (C), 128.42 (C), 127.19 (2CH), 125.79 (2CH), 123.55 (CH), 122.50 (CH), 117.68 (CH), 111.16 (CH), 33.46 (CH), 22.99 (2CH_3_). ESI-MS (m/z):calcd. for C_16_H_16_N_2_ 237.1386, found 237.1388 [M+H]^+^.

### 3.2. 5-Chloro-2-(4-Isopropylphenyl)-1H-Benzo[d]imidazole (**2a**)

Compound **2a** (C_16_H_15_ClN_2_, MW 270.76) was obtained in a total yield of 75%; m.p. 228-230 °C; TLC (PE/EA 7/3): R_f_ 0.75. ^1^H-NMR (DMSO-d_6_): δ 8.12 (2H, d, J = 8.4 Hz, H-2′,6′), 7.66 (1H, d, J = 1.6 Hz, H-4), 7.61 (1H, d, J = 8.8 Hz, H-7), 7.40 (2H, d, J = 8.4 Hz, H-3′,5′), 7.19 (1H, dd, J_ortho_ = 8.6 Hz, J_meta_ = 1.6 Hz, H-6), 2.95 (1H, q, aliphatic CH), 1.24 (6H, d, J = 6.8 Hz, 2CH_3_). ^13^C-NMR (jmod, DMSO-d_6_): δ 152.90 (C), 150.61 (C), 127.46 (2C), 126.86 (2CH), 126.67 (2CH), 126.06 (2C), 121.97 (CH), 116.01 (CH), 114.85 (CH), 33.32 (CH), 15.18 (2CH_3_). ESI-MS (m/z): calcd. for C_16_H_15_ClN_2_ 271.0997, found 271.1000 [M+H]^+^.

### 3.3. 5,6-Dichloro-2-(4-Isopropylphenyl)-1H-Benzo[d]imidazole (**3a**)

Compound **3a** (C_16_H_14_Cl_2_N_2_, MW 305.202) was obtained in a total yield of 87%; m.p. 257-259 °C; TLC (PE/EA 7/3): R_f_ 0.75. ^1^H-NMR (DMSO-d_6_): δ 8.19 (2H, d, J = 8 Hz, H-2′,6′), 7.96 (2H, s, H-4,7), 7.53 (2H, d, J = 8 Hz, H-3′,5′), 3.01 (1H, q, J = 6.8 Hz, CH), 1.26 (6H, d, J = 6.8 Hz, 2CH_3_). ^13^C-NMR (jmod, DMSO-d_6_): δ 153.19 (C), 152.38 (C), 135.09 (2C), 127.70 (2CH), 127.60 (2CH), 126.43 (2C), 123.42 (C), 115.73 (2CH), 33.47 (CH), 23.46 (2CH_3_). ESI-MS (m/z): calcd. for C_16_H_14_Cl_2_N_2_ 305.0607, found 305.0608 [M+H]^+^. 

### 3.4. 5-Fluoro-2-(4-Isopropylphenyl)-1H-Benzo[d]imidazole (**4a**)

Compound **4a** (C_16_H_15_FN_2_, MW 254.302) was obtained in a total yield of 76%; m.p. 255 °C; TLC (PE/EA 7/3): R_f_ 0.56. 1H-NMR (acetone-d_6_): δ 8.14 (2H, d, J = 8 Hz, H-2′,6′), 7.58 (1H, m, H-4), 7.44 (2H, d, J = 8 Hz, H-3′,5′), 7.32 (1H, d, J_ortho_ = 8 Hz), 7.02 (1H, ddd, J_para_ = 1.8 Hz, J_ortho_ = 8Hz, ^3^J_H-F_ = 11.4 Hz, H-6), 3.01 (1H, q, J = 7 Hz, aliphatic-CH), 1.30 (6H, d, J = 7.2 Hz, 2CH_3_). ^13^C-NMR (APT, DMSO-d_6_): δ 159.78 (C), 157.44 (C), 152.72 (C), 150.80 (C), 137.47 (1C, d, ^1^J_C-F_ = 402 Hz, C-F), 127.12 (C), 126.96 (2CH), 126.50 (2CH), 115.45 (CH-7), 110.17 (1C, d, ^2^J_C-F_ = 26 Hz, CH-6), 100.99 (1C, d, ^2^J_C-F_ = 25 Hz, CH-4), 33.29 (CH), 23.56 (2CH_3_). ESI-MS (m/z): calcd. for C_16_H_15_FN_2_ 255.1292, found 255.1289 [M+H]^+^.

### 3.5. 5,6-Difluoro-2-(4-Isopropylphenyl)-1H-Benzo[d]imidazole (**5a**)

Compound **5a** (C_16_H_14_F_2_N_2_, MW 272.293) was obtained in a total yield of 67%; m.p. 235–237 °C; TLC (PE/EA 7/3): R_f_ 0.75. ^1^H-NMR (DMSO-d_6_): δ 8.17 (2H, d, J = 8.4 Hz, H-2′,6′), 7.82 (2H, t, J = 8.8 Hz, H-4,7), 7.54 (2H, d, J = 8 Hz, H-3′,5′), 3.01 (1H, m, J = 6.8 Hz, CH), 1.26 (6H, d, J = 6.8 Hz, 2CH_3_). ^13^C-NMR (jmod, DMSO-d_6_): δ 153.24 (C), 151.67 (C), 147.94 (2C, dd, ^1^J_C-F_ = 242 Hz, ^2^J_C-F_ = 16 Hz, 2CF),130.26 (2C), 127.57 (2CH), 127.41 (2CH), 122.94 (C), 102.52 (2CH, m, CH-4,7), 33.48 (CH), 23.45 (2CH_3_). ESI-MS (m/z): calcd. for C_16_H_14_F_2_N_2_ 273.1198, found 273.1195 [M+H]^+^.

### 3.6. 2-(4-(Tert-Butyl)phenyl)-1H-Benzo[d]imidazole (**1b**)

Compound **1b** (C_17_H_18_N_2_, MW 250.338) was obtained in a total yield of 65%; m.p. 239-241 °C; TLC (PE/EA 7/3): R_f_ 0.50. ^1^H-NMR (DMSO-d_6_): δ 8.11 (2H, d, J = 8.4 Hz, H-2′,6′), 7.65 (1H, d, J = 7.2 Hz, H-4), 7.57 (2H, d, J = 8.4 Hz, H-3′,5′), 7.52 (1H, d, J = 6.8 Hz, H-7), 7.19 (2H, m, H-5,6), 1.34 (9H, s, 3CH_3_). ^13^C-NMR (jmod, DMSO-d_6_): δ 152.53 (C), 151.24 (C), 143.82 (C), 134.92 (C), 127.41 (C), 126.19 (2CH), 125.70 (2CH), 122.29 (CH), 121.50 (CH), 118.69 (CH), 111.16 (CH), 34.56 (C), 30.96 (3CH_3_). ESI-MS (m/z): calcd. for C_17_H_18_N_2_ 251.1543, found 251.1544 [M+H]^+^.

### 3.7. 2-(4-(Tert-Butyl)phenyl)-5-Chloro-1H-Benzo[d]imidazole (**2b**)

Compound **2b** (C_17_H_17_ClN_2_, MW 284.783) was obtained in a total yield of 83%; m.p. 247–249 °C; TLC (PE/EA 7/3): R_f_ 0.76. ^1^H-NMR (acetone-d_6_): δ 8.17 (2H, d, J = 8.4 Hz, H-2′,6′), 7.62–7.59 (4H, m, H-4,7,3′,5′), 7.23 (1H, dd, ^1^J = 8.6 Hz, ^2^J = 1.6 Hz, 2 Hz, H-6), 1.38 (9H, s, 3CH_3_). ^13^C-NMR (jmod, acetone-d_6_): δ 154.37 (C), 153.81 (C), 141.61 (C), 139.11 (C), 128.10 (C), 128.07 (C), 127.40 (2CH), 126.75 (2CH), 126.35 (CH), 116.83 (CH), 115.80 (CH), 35.45 (C), 31.45 (3CH_3_). ESI-MS (m/z): calcd. for C_17_H_17_ClN_2_ 285.1153, found 285.1157 [M+H]^+^.

### 3.8. 2-(4-(Tert-Butyl)phenyl)-5,6-Dichloro-1H-Benzo[d]imidazole (**3b**)

Compound **3b** (C_17_H_16_Cl_2_N_2_, MW 319.228) was obtained in a total yield of 63%; m.p. 259–261 °C; TLC (PE/EA 7/3): R_f_ 0.84. ^1^H-NMR (DMSO-d_6_): δ 8.20 (2H, d, J = 8.4 Hz, H-2′,6′), 7.96 (2H, s, H-4,7), 7.68 (2H, d, J = 8.4 Hz, H-3′,5′), 1.35 (9H, s, 3CH_3_). ^13^C-NMR (jmod, DMSO-d_6_): δ 154.82 (C), 152.88 (C), 136.28 (2C), 127.12 (2CH), 126.15 (2CH), 125.82 (2C), 124.09 (C), 115.89 (2CH), 34.81 (C), 30.80 (3CH_3_). ESI-MS (m/z): calcd. for C_17_H_16_Cl_2_N_2_ 319.0763, found 319.0766 [M+H]^+^.

### 3.9. 2-(4-(Tert-Butyl)phenyl)-5-Fluoro-1H-Benzo[d]imidazole (**4b**)

Compound **4b** (C_17_H_17_FN_2_, MW 268.329) was obtained in a total yield of 84%; m.p.> 300 °C; TLC (PE/EA 7/3): R_f_ 0.72. 1H-NMR (DMSO-d_6_): δ 8.09 (2H, d, J = 8 Hz, H-2′,6′), 7.57 (3H, d, J = 8.4 Hz, H-4,3′,5′), 7.38 (1H, d, J = 8.4 Hz, H-7), 7.05 (1H, m, H-6), 1.33 (9H, s, 3xCH_3_). ^13^C-NMR (jmod, DMSO-d_6_): δ 159.83 (C), 157.50 (C), 153.22 (C), 152.60 (C), 137.22 (1C, d, ^1^J_C-F_ = 401 Hz, C-F),126.44 (C), 126.28 (2CH), 125.87 (2CH), 115.50 (1C, d, ^3^J_C-F_ = 10 Hz, CH-7), 110.38 (1C, ^2^J_C-F_ = 25 Hz, CH-6), 100.95 (1C, ^2^J_C-F_ = 26 Hz, CH-4), 34.55 (C), 30.84 (3CH_3_). ESI-MS (m/z): calcd. for C_17_H_17_FN_2_ 269.1448, found 269.1448 [M+H]^+^.

### 3.10. 2-(4-(Tert-Butyl)phenyl)-5,6-Difluoro-1H-Benzo[d]imidazole (**5b**)

Compound **5b** (C_17_H_16_F_2_N_2_, MW 286.319) was obtained in a total yield of 78%; m.p. 257-259 °C; TLC (PE/EA 7/3): R_f_ 0.78. ^1^H-NMR (DMSO-d_6_): δ 8.14 (2H, d, J = 8.4 Hz, H-2′,6′), 7.78 (2H, t, J = 8.8 Hz, H-4,7), 7.66 (2H, d, J = 8.4 Hz, H-3′,5′), 1.35 (9H, s, 3CH_3_). ^13^C-NMR (jmod, DMSO-d_6_): δ 154.65 (C-2), 152.25 (C-4′), 147.56 (2C, dd, ^1^J_C-F_ = 241 Hz, ^2^J_C-F_ = 16 Hz, 2CF), 131.75 (C-3a,7a), 126.86 (C-2′,6′), 126.15 (C-3′,5′), 123.97 (C-1′), 102.66 (2CH, dd, ^2^J_C-F_ = 15 Hz, CH-4,7), 34.80 (C), 30.81 (3CH_3_). ESI-MS (m/z): calcd. for C_17_H_16_F_2_N_2_ 287.1354, found 287.1359 [M+H]^+^.

## 4. Results

### 4.1. Chemistry

**Scheme 1:**
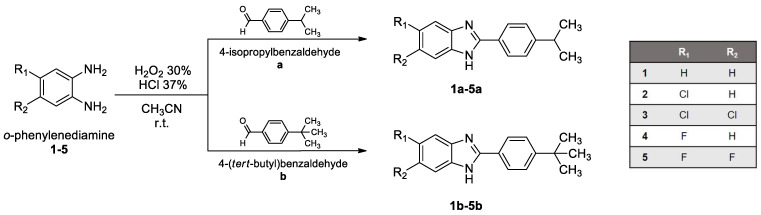
**1a**,**b**-**5a**,**b**.

Benzimidazole derivatives were designed and synthesized as reported in Scheme 1. o-Phenylenediamine (**1-5**) and the corresponding aldehyde (**a** and **b**) were mixed and reacted in a single-pot, simple condensation reaction at room temperature, obtaining derivatives **1a**,**b**-**5a**,**b**.

### 4.2. Antiviral Assays

All newly synthesized 2-phenyl-benzimidazole derivatives were evaluated for their broad-spectrum anti-enterovirus activity in cell-based assays. Several compounds exhibited significant inhibitory activity, with EC_50_ values in the low micromolar range (i.e., <10 µM, Table 1). Concomitantly, moderate to low cytotoxicity was detected for almost all compounds, with CC_50_ values mostly in the high micromolar range (>100 µM) in Vero-76 cells. Only **1a**,**b**, and **3a,b** showed cytotoxicity in the low micromolar range against Vero-76, HeLa, and LLC-MK2 cell monolayers.

Concerning the spectrum of antiviral activity, the most relevant results referred to the interesting activity of derivatives **2b** and **2a** against the broad panel of enteroviruses (Table 1). 

Compound **2a** showed remarkable antiviral activity against Entero A, B, and C viruses with an EC_50_ lower or equal to 10 µM. No activity was detected against EV-D68 (>100 µM) or CV-B4. The comparable interesting activity was determined for compound **2b** against all the Entero strains tested. The same lack of activity was described for EV-D68 (>100 µM). Particularly, **2b** resulted in being effective in reducing cytopathy induced by EV-A71 with an EC_50_ of 3 µM and a selectivity index > 30. Further, derivatives **5a** and **5b** resulted in being active against Poliovirus 1 (PV-1) and EV-A71 with an EC_50_ range of 12–31 and 10–6 µM, respectively.

Compound **5b** also showed an interesting EC_50_ against EV-D68 (4 µM). Compound **1b** resulted in being active against CV-B5 and PV-1 with an EC_50_ range of 7–9 µM, respectively, while derivative **1a** was endowed withmoderate activity against PV-1 (EC_50_ = 11.5 µM). Compounds **3a** and **3b** resulted in being cytotoxic and weakly active (**3b** CC_50_ = 9 µM and EC_50_ = 2 µM) against CV-B5. 

From a structure–activity relationship (SAR) perspective, we can state the relevance of the substitution of the benzimidazole (BIZ) scaffold since derivatives **1a** and **1b**, with no substituents, are more endowed with cytotoxicity than with antiviral activity. At the same time, we detected high toxicity also when BIZ was substituted with two bigger chlorine atoms in positions **5** and **6** (series **3**). In terms of cytotoxicity, the substitution with a moderate steric hindrance such as one bigger chlorine atom (series **2**) and one or two smaller fluorine atoms (series **4** and **5**) turned out as the best option. Non-toxic series **2** and **5** are also complemented with the best antiviral activity detected. Interestingly, the presence of one single chlorine atom on the BIZ scaffold provided series **2** derivatives a wider antiviral activity, as can be seen from Table 1, showing remarkable EC_50_ values when tested against EV-A71, CV-B3, CV-B5, PV-1, and also Echo 9, which was successfully inhibited only by the derivatives **2a** and **2b**.

Regarding series **5**, two fluorine atoms were placed in positions **5** and **6** on BIZ, whichendowed derivatives **5a** and **5b** withmore selective antiviral activity. Series **5** showed relevant EC_50_ values when tested against PV-1, EV-A71, and EV-D68 (**5b**). To evaluate the weight of the sterical hindrance on position 4′ of the phenyl substituent, we selected isopropyl (**a**) and tert-butyl moieties (**b**) for the purpose. It is clearly shown that the most hindered tert-butyl moiety lent an increased activity profile if compared with the parental derivative which brings the isopropyl substituent on the same 4′ position. Focusing on EV-A71, compound **2b** presented an EC_50_ value of 3.5 µM, while the parental **2a** value was recorded at 7 µM. It is even more striking when series 5 is considered, where 5b showed an EC_50_ value of 6 µM, while **5a** showed only 31 µM. Derivative **5b** is also quite potent when inhibiting EV-D68 replication at 4 µM concentration, while the parental **5a** is not active when tested against this Enterovirus. It can be concluded that the tert-butyl moiety provides the best antiviral activity to benzimidazoles that present one big or two small halogen atoms on the BIZ scaffold. 

Based on the described results, **2b** showed the most compelling profile, resulting in a promising antiviral activity against EV-A71 and extendedspectrum against CV-B3, CV-B4, CV-B5, Echo 9, and PV-1.Concomitantly, the general low cytotoxic profile of **2b** was proved in the cell lines, sustaining the viral replication of several strains (Table 2).

Starting from these considerations and taking into account the recent EV-A71 epidemics that occurred in Europe, the concern about the potential emergence of EV-A71 as a worldwide health threat, and the lack of an established treatment, we then decided to select compound **2b** and EV-A71 for additional investigations, as described in detail below.

### 4.3. Protective Effect of **2b** on EV-A71 Infected Cells

**Figure 1:**
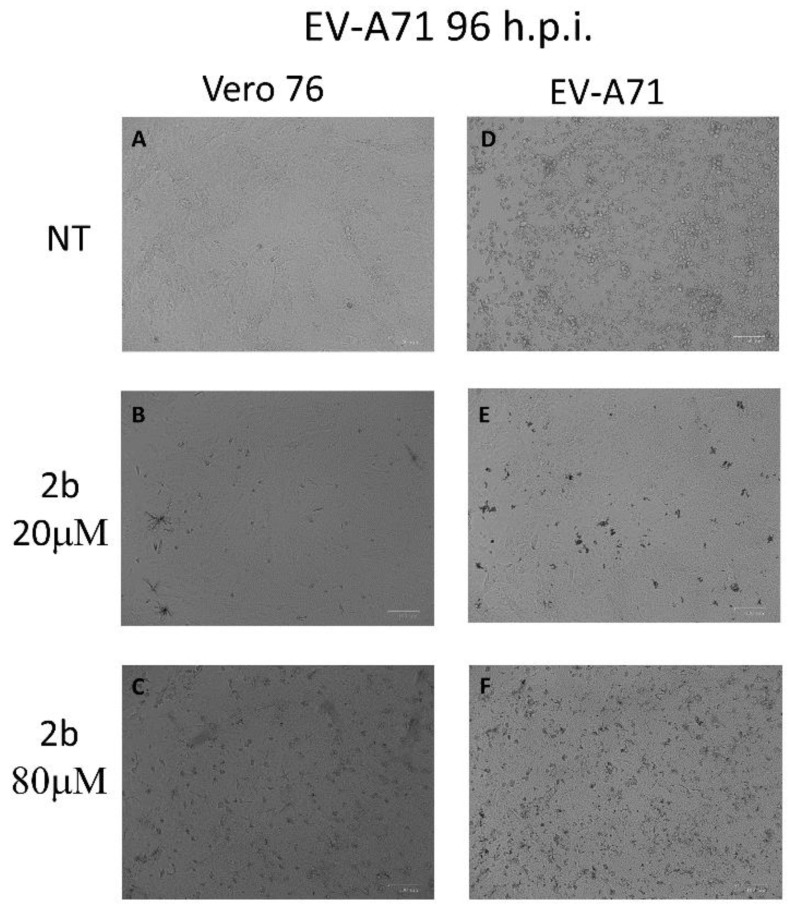
Effect of **2b** inhibitor (20 and 80 µM) on the Vero-76-infected monolayers. Control (**A**), treated cells with 20 µM (**B**), treated cells with 80 µM (**C**), infected cells (**D**), infected treated cells (20 µM) (**E**), and infected treated cells (80 µM) (**F**). Pictures of cell morphology were taken at 72 h post-infection using ZOE Fluorescent cell imager (Bio-Rad) (bar size = 100 μm, magnification, 20×).

To verify whether derivative **2b** could interfere in EV-A71-induced apoptosis and preserve the vitality of monolayers, Vero-76 cells, growth in 12-well plates, were untreated or infected with an m.o.i. of 0.1 of EV-A71. After viral adsorption, the cells were incubated in the absence or presence of 20 and 80 µM of **2b**. As shown in Figure 1, the intact cell monolayer suggests that both concentrations displayed no cytotoxic effect against cells (Figure 1A–C), whereas, after infection, EV-A71 showed a cytopathic effect with morphological variations of monolayer and round-shaped cells representing the dead cells (Figure 1D). On the contrary, treatment with 20 µM and 80 µM of the compound seems to protect cells from EV-A71 infection and no evident cytotoxic effects were detected up to a concentration of 80 µM. (Figure 1E,F). To validate this morphological result, the cells were stained with Annexin-V-fluorescein and propidium iodide and subsequently subjected to flow cytometry analysis.

**Figure 2:**
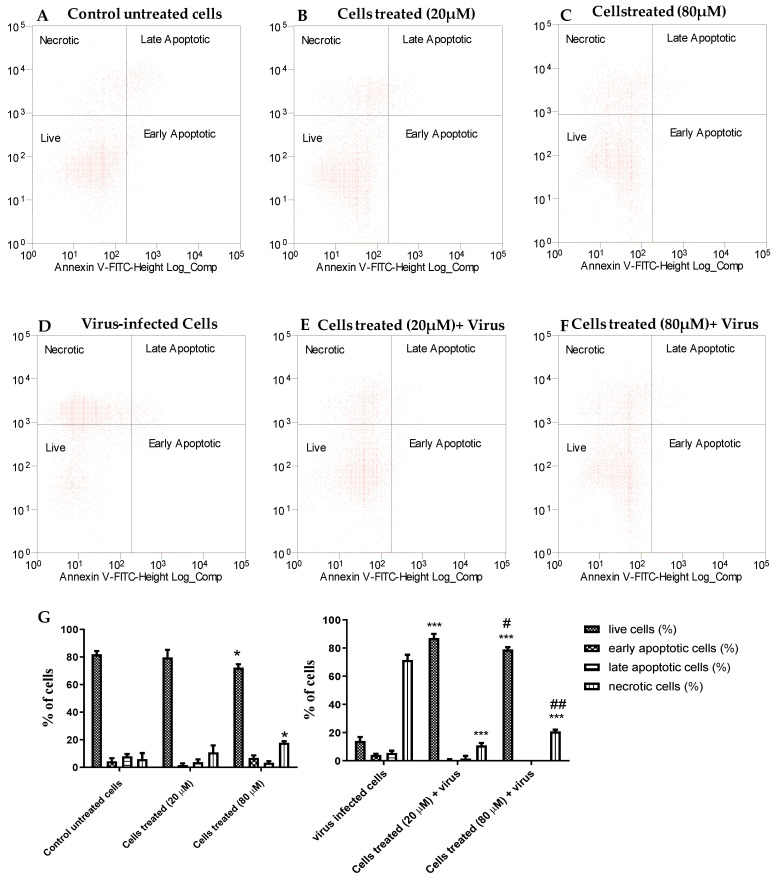
The inhibitory effect of compound **2b** on EV-A71-induced apoptosis. The percentage of live, apoptotic, and necrotic cells were measured by flow cytometry using the PI-annexin V assay. Dot plots show cell death in Vero-76 cells: control (**A**), treated cells with 20 µM (**B**), treated cells with 80 µM (**C**), infected cells (**D**), infected treated cells (20 µM) (**E**), and infected treated cells (80 µM) (**F**). Percentage of live, apoptotic, and necrotic cells (**G**). Statistically significant differences are expressed as follows: * = *p*< 0.05 vs. untreated cells; *** = *p*< 0.001 vs. untreated cells; # = *p*< 0.05 vs. cells treated (20 µM) + virus; ## = *p*< 0.01 vs. cells treated (20 µM) + virus.

The dot plots in Figure 2 show the Vero-76 cell distribution within four different cell populations: live, apoptotic, late apoptotic, and necrotic cells, and represent one of three sets of independent experiments conducted. In the untreated cells, there was a minimal number of necrotic, early, and late apoptotic cells and this trend was very similar to the 20 µM-treated cells (Figure 2A,B), whereas necrotic cells slightly increased in treated cells with 80 µM concentration if compared to untreated cells (Figure 2C), confirming the minimal cytotoxic effect of our derivative. The A71 virus is able to induce necrosis in about 70% of untreated cells, while compound **2b** effectively reduces the death of Vero-76 cells. When treated with 20 and 80 µM, EV-A71-infected cells are significantly protected from necrosis and apoptosis, displaying 90% and 80% of viability and only 10% and 20% of necrotic cells, respectively (Figure 2E–G).

### 4.4. TEER Experiment

**Figure 3:**
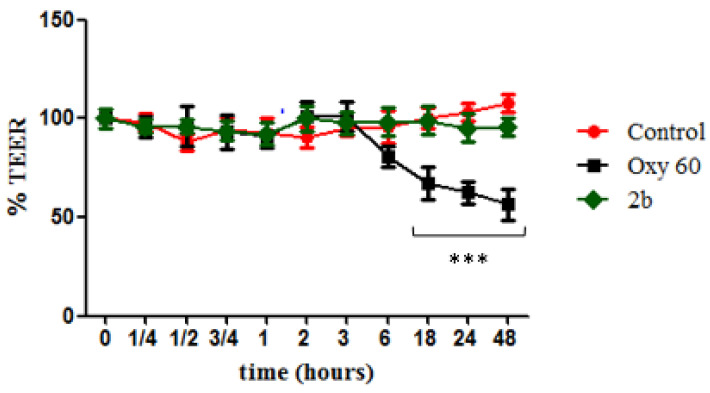
Measurement of cell monolayer permeabilization (transepithelial electrical resistance (TEER)) assay. Caco-2 cell monolayers were incubated with OXY at 60 µM (black squares) as negative control, derivative **2b** at 20 µM (blue triangles), and Control (red circles) as internal positive control. Statistically significant differences are expressed as follows: *** = *p* < 0.001 vs. Control. Each value represents the mean ± SD of independent experiments (*n* = 3).

To ascertain any toxic effect of derivative **2b** on human cells, we tested it on differentiated intestinal Caco-2 monolayers, commonly used to simulate the gut epithelium and to evaluate changes in intestinal permeability. Cells were treated with an oxysterols mixture (Oxy, 60 µM) as a negative control, and it was observed that it caused cellular redox imbalance, reflected in a significant alteration of the monolayer integrity with time (Figure 3), starting from 18 h of incubation, when the TEER value was about 65% of the level of the untreated cells. TEER values measured in monolayers treated with compound **2b** (20 µM) were instead not significantly different from control values throughout all the time points, showing no enhancement of cell permeability. 

### 4.5. Yield Reduction Assay and Study of the Mechanism of Action

**Figure 4:**
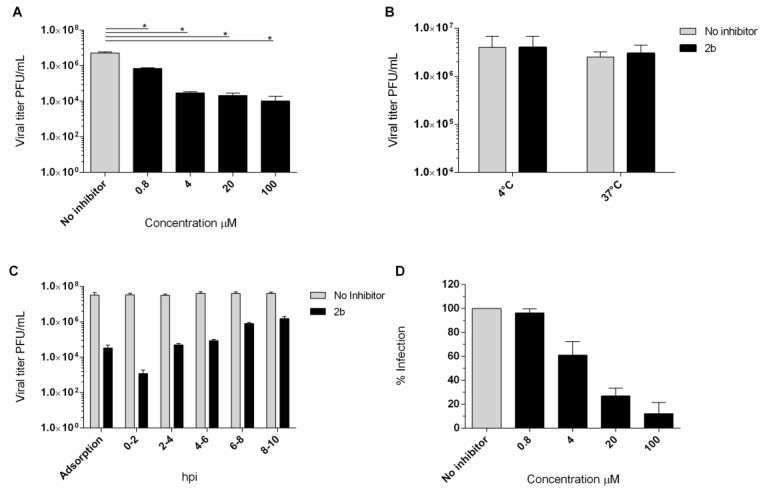
(**A**) Yield of infectious EV-A71 viruses produced in infected Vero-76 cells treated with 2b and NM 107. Vero-76 cells were infected with EV-A71 (m.o.i. 0.1). The infected cultures were treated with **2b**, at indicated doses. Viral yields in the culture supernatant were determined by a plaque assay at 96 h post-infection. * Statistically significant differences are expressed (*p*< 0.05). (**B**) Virucidal effect (expressed as plaque-forming units (PFU/mL) of derivative **2b** (20 µM) against EV-A71 virions at either 4 °C or 37 °C for 1 h. Dark columns, viral titer for viral and derivative **2b** solution; white columns for the viral titer of the untreated solution. (**C**) Adsorption assay and time course experiment. Vero-76 cells were pre-adsorbed for 1 h at 4 °C with viruses at an m.o.i. = 1 in the presence of the compound (5 × EC_50_ concentration). Vero-76 cells were inoculated with EV-A71 (m.o.i. = 1) and then compound **2b** (20 µM) was added at the indicated times. Viral yields were determined by a plaque assay. Dark columns, the viral yield for control cells; gray columns; viral yield for cells treated with **2b** derivative. (**D**) Penetration assay. Dose-dependency of derivative **2b** (dark columns) in reduction in EV-A71 penetration compared to non-treated sample control (gray columns). The results presented were obtained from three independent experiments. Data are mean ± SD.

The antiviral activity of compound **2b** was investigated in a yield reduction assay, in order to ascertain the reduction invirus titer in the presence of the active compound. Non-cytotoxic concentrations of 100, 20, 4, and 0.8 µM were used and a dose-dependent reduction inthe titer was observed. An important reduction in theEV-A71 titer was detected also in a low concentration (Figure 4A). To assess the effect of compound **2b** on viral infectivity, a virucidal assay was performed. No significant differences between the titers of EV-A71 treated at the two different temperatures were observed (Figure 4B). Compound **2b** failed to affect the EV-A71 infectivity, suggesting that the inhibition of EV-A71 replication observed in cell-based assays could be due to interference with a step along the EV-A71 replication cycle. Furthermore, no inhibition was observed when cells were pre-incubated (2 h) with 20 µM concentration of derivative **2b** and then infected with untreated EV-A71 (data not shown).

The kinetics of virus adsorption in the presence of derivative **2b** was also evaluated. Low-temperature treatment permits the binding of viruses to the cell surface receptors but avoids the internalization of viral particles into the host cells. Accordingly, Vero-76 cells were incubated with EV-A71 (m.o.i. = 1) and compound **2b** for 2 h at 4 °C, using compound concentrations of 20 µM. The treatment with compound **2b** resulted in a detectable reduction in the virus titer in comparison to the untreated infected control (Figure 4C). Furthermore, we assessed the potential mode of antiviral activity with a time-of-addition assay (Figure 4C). The experiment was performed in EV-A71-infected Vero-76 cells (m.o.i. = 1) exposed to the compound (20 µM) at different times of infection. Interestingly, Figure 4C showed that the compound exerted the greatest effect when added along with the virus. Moderate titer reduction is detectable until 2 h post-infection. However, a reduction in virus yield was detected if compared to the untreated control, even when the compound was added at the late stages of the growth cycle. 

We performed a penetration assay to scan the mechanism of inhibition of the entry of EV-A71 by compound **2b** into Vero-76 cells. The percentage of reduction in viral penetration was determined in relation to the untreated control.We found that compound **2b** could effectively inhibit the viral penetration in a dose-dependent manner with an EC_50_ of 6.2 µM ± 2. (Figure 4D).

### 4.6. Druglikeness, Pharmacokinetic and Toxicity Predictions

Theoretical druglikeness, pharmacokinetic, and toxicity properties prediction is a key step between in vitro and in vivo experiments. The PreADMET (http://preadmet.bmdrc.kr/) and SwissADME (http://www.swissadme.ch/) tools were employed to assess the newly synthesized library of compounds (**1a**-**5a**, **1b**-**5b**) and results are reported in Table 3. On the base of Lipinski’s“rule of five”, all the tested compounds are suggested to be drug-like molecules. According to pharmacokinetic predictions, compounds are all strong binders to the plasma protein with %plasma protein binding (PPB) values higher than 90%. They are all suggested to be absorbed into the CNS with high BBB (blood–brain barrier) permeability scores. All molecules are supposed to be moderately water-soluble. Lipophilicity is predicted to be good in general terms, with CLog Po/w values ranging between 3.88 and 5.19. As a matter of fact, gastrointestinal absorbance of these compounds is expected to be from middle to high. With the sole exception of compounds **1a**, **4a,** and **5a**, they are generally considered not to be a substrate for P-glycoprotein efflux. In order to assess whether these new molecules could be used for in vivo assays, a toxicity profile prediction was performed using the PreADMET tool. Most of our compounds were mutagenic in the Ames test either with or without metabolic activation, except for our lead compound **2b** and derivative **3b**. The human ether a go-go-related (hERG) gene encodes cardiac potassium channels, inhibition of which would prolong the QTc interval along with the risk of cardiac arrhythmias [29]. Toxicity predictions resulted in medium risk of hERG inhibition for our compounds. Before starting with eventualin vivoexperiments, it will be necessary to assess the concentration at which the hERG inhibition rate is troubling and compare it with the EC_50_ values fromthe in vitroanalysis. All considered, the newly designed and synthesized compounds showed good properties of predicted pharmacokinetics and druglikeness, our lead compound **2b** was proved to be a drug-like molecule, with the lowest toxicity profile coupled with discrete water solubility and a good lipophilicity property. Absorption and distribution are predicted to be valuable. Hence, compound **2b** could be a good candidate for further in vivo assays.

## 5. Discussion

In this study, we describe the promising anti-enteroviral activity of a selected group of new benzimidazoles. Among them, derivative **2b** turned out to be endowed with a considerable broad-spectrum anti-enteroviral activity with a cytotoxic profile in the high micromolar range (Table 1). We documented the remarkable antiviral activity against the strain BrCr of EVA71, genogroup A.

This strainwas firstly isolated from a stool specimen of a patient with acute meningitis and from the brain of a patient who died of encephalitis [30]. This virus is a causal agent of severe CNS diseases, and for this reason, it was employed in our assays as a prototype and reference strain of EVA71.

To validate the suitability of compound **2b** and to rule out its toxicity against human epithelial cells, its impact on Caco-2 cell monolayers permeability was evaluated. Caco-2 cells, coming from the human colonic epithelium, fully differentiate to enterocytes in vitro and are commonly used to evaluate the effect of nutrients as well as contaminants and drugs [31].

We tested compound **2b** at 20 μM (active and non-cytotoxic concentration), and it was observed that it did not induce any significant change in permeability when compared to untreated cells, up to 48 h (the longest time of incubation). Therefore, it did not exert any toxic effects on epithelial cells, resulting as safe at the tested concentration. Derivative **2b**, inter alia, was proved to be not only able to protect cell monolayers from EV-A71-induced cytopathogenicity, with an EC_50_ value of 3 µM, but also Vero-76 cells when treated with 20 and 80 µM of **2b**, also resulting as being significantly protected from cell death (Figure 2). Generally, it has been demonstrated that EV-A71 can induce apoptosis [32,33]. In our hands, EV-A71 infection seems not to induce apoptosis but rather 70% of Vero-76 cells died from other forms of cell death such as necrosis or pyroptosis. Pyroptosis is a novel form of programmed cell death and an increasing number of studies have demonstrated that different viral infections [34,35,36], including EV-A71 infections, may activate this death pathway [37,38,39,40]. We can hypothesize that long-time (96 h, needed to reach optimal CPE) virus exposure leads to a switch from apoptosis to necrosis or that the pyroptosis process is involved. In any case, accurate investigations are needed. However, our goal was to understand whether the cells treated with **2b** were protected from infection and, in parallel, to validate results obtained from the antiviral assay. Thus, we demonstrated that compound **2b**, at both tested concentrations, can preserve the cells from death. In this study, we also showed that **2b** was able to reduce the viral titer in a dose-dependent manner. Cells pretreated with **2b** did not show inhibition of A71 replication, indicating that **2b** does not act on cell receptors, as well as the treatment of viral particles not being able to determine the direct inactivation of the virion. Furthermore, we demonstrated that **2b** reduces viral adsorption and penetration to Vero-76 cells by the time-of-addition assay and penetration assay when compared to untreated samples. Our derivative had its utmost effect when added during the infection period. Based on all these results, we can hypothesize that our compound can inhibit viral endocytosis by reducing viral attachment to and penetration into Vero monolayers. Compound **2b** is endowed with low cytotoxicity against cell monolayers supporting the replication of EV-A71 but also against a large group of cell cultures, as reported in Table 2. 

These results need to be interpreted in light of the strengths and limitations of our study. Some of the major strengths of our study are the characterization of a new class of compounds with a broad-spectrum anti-enterovirus activity and the identification of derivative **2b**, which is endowed with remarkable anti-EV A71 activity. Preliminary mode of action studies showed that **2b** exerts its activity during an early step of the viral cycle, also reducing the penetration phase. Moreover, the ADME and toxicity prediction of our derivatives suggested that **2b** is endowed withlow toxicity, good lipophilicity, and discrete water solubility. Pharmacokinetic predictions showed that our lead compound can be easily absorbed into the CNS with high BBB permeability scores. This is an important prerequisite for the development of molecules that could take effect in the CNS. Since, at present, there is no effective treatment against EV-A71, maybe because many tested compounds failed due to a lack of the ability to penetrate the BBB, and monovalent vaccines are currently limited to the Chinese regions, this derivative could be considered as a good starting point for the development of effective candidates for treatment against EVA71. Considerable efforts have been made, by numerous scientists, to reproduce the human pathology induced by EV-A71 in animals such as cynomolgus and rhesus monkeys, as well as in laboratory mice and other mammals [41,42,43,44,45,46,47,48]. 

However, existing animal models of Enterovirus A71 infection do not reproduce, in animals, the complete spectrum of neurological hallmarks detected in humans [49] and the use of nonhuman primates as animal models is very tricky because of the ethical and cost problem.Another limit is that we documented antiviral activity against the strain BrCr of EVA71, genogroup A, and it would be important to validate our results against genotypes B and C, which are characterized by a wider diffusion [8]. 

That considered, further studies should be carried out to understand which animal model could be the most suitable to reproduce human EV-A71 neurological features. Meanwhile, it is necessary to clarify the mechanism of antiviral action of this promising compound.

## Figures and Tables

**Table 1:** Cytotoxicity and antiviral activity of benzimidazole (BIZ) derivatives and references against representatives of Enterovirus (EV-A71, CV-B3, CV-B4, CV-B5, PV-1, EV-D68, and Echo9).

**Cmp**	Vero-76	EV-A71	CV-B3	CV-B4	CV-B5	PV-1	HeLa	EV-D68	LLC-MK2	Echo9
	^a^ CC_50_	^b^ EC_50_	^c^ EC_50_	^c^ EC_50_	^c^ EC_50_	^c^ EC_50_	^d^ CC_50_	^c^ EC_50_	^e^ CC_50_	^c^ EC_50_
**1a**	60/24 *	>24	>60	>60	>60	11.5	9	>9	6	>6
**1b**	46/40 *	>40	>46	>46	**7**	**9**	9	>9	6.5	>6.5
**2a**	>100	**7**	17	≥100	**10**	**9**	100	>100	>100	**8**
**2b**	>100	**3.5**	33	25.5	**10**	**6**	100	>100	12	**4**
**3a**	25.5/8 *	>8	>25.5	>25.5	>25.5	>25.5	8	>8	7	>7
**3b**	9	>9	>9	>9	**2**	**-**	6.4	>6.4	12	>12
**4a**	>100	>100	>100	>100	>100	≥100	>100	>100	29.5	>29.5
**4b**	>100	70	>100	>100	>100	**20**	>100	>100	>100	>100
**5a**	>100	**31**	>100	>100	>100	**12**	>100	>100	100	>100
**5b**	>100	**6**	>100	>100	>100	**10**	>100	**4**	>100	>100
**Plc**	>100	0.005 ± 0.001	2 ± 0.005		2 ± 0.5			0.3	>100	0.1
**NM 107**	>100			29		6				
**Rup**	>100					0.07				

^a–e^ Compd conc (µM) required to reduce by 50% the viability of mock-infected Vero-76, HeLa, and LLC-MK2, as determined by the MTT method after 3 * days, 4 days, and 5 days, respectively. ^b^ Compd conc (µM) required to achieve 50% protection of Vero-76 or HeLa cells from EV-A71- and EV-D68-induced cytopathogenicity, as determined by the MTT method at days 3 and 4-5 p.i., respectively. ^c^ Compd conc (µM) required to reduce by 50% the plaque number of CV-B3, CV-B4, CV-B5, PV-1,and Echo9 in Vero-76 and LLC-MK2 cells. Plc (Pleconaril); NM107 (2′-C-methylcytidine); Rup (Rupintrivir). Data represent mean values + SD for three independent determinations. For values where SD is not shown, variation among samples was less than 15%.

**Table 2:** Cytotoxicity of benzimidazole (BIZ) derivatives against selected cell lines °.

Compounds	BHK-21	MDBK	Vero-76	HeLa	LLC-MK2
	^a^ CC_50_	^b^ CC_50_	^c^ CC_50_	^d^ CC_50_	^e^ CC_50_
**1a**	36	65	60/24 *	9	6
**1b**	20	9.0	46/40 *	9	6.5
**2a**	>100	80	>100	100	>100
**2b**	>100	>100	>100	100	12
**3a**	8.0	2.5	25.5/8 *	8	7
**3b**	9	7	9	6.4	12
**4a**	>100	>100	>100	>100	29.5
**4b**	>100	>100	>100	>100	>100
**5a**	>100	>100	>100	>100	100
**5b**	>100	>100	>100	>100	>100

° Data represent mean values + SD for three independent determinations. For values where SD is not shown, variation among samples was less than 15%. ^a–e^ Compound conc (µM) required to reduce by 50% the viability of mock-infected BHK-21, MDBK, Vero-76, HeLa, and LLC-MK2, as determined by the MTT method after 3 * days, 4 days, and 5 days, respectively.

**Table 3:** Absorption, distribution, metabolism, excretion(ADME)and toxicity prediction of compounds (**1a**-**5a**, **1b**-**5b**).

	*ADME Profile*									*Toxicity ^a^*					
Samples	PPB (%) ^a^	BBB Permeability			Lipophilicity ^b^	Water sol ^b^	GI abs ^b^	Caco-2 cells ^a^	P-gp sub ^b^	hERG Inhibition	Ames Test				
		(C_brain_/C_blood_) ^a^	Pr. ^a^	Pr. ^b^	Consensus	Solubility		(nm/sec)			Pr.	TA100		TA1535	
					Log *P_o/w_*	Class						S9 act	No S9 act	S9 act	No S9 act
**1a**	92	11.95	High	Yes	3.88	Soluble (m)	High	36.9	Yes	Medium	M	Pos	Neg	Neg	Pos
**1b**	95	12.61	High	Yes	4.14	Soluble (m)	High	42.3	No	Medium	M	Pos	Neg	Neg	Neg
**2a**	90	13.29	High	Yes	4.41	Soluble (m)	High	53.2	No	Medium	M	Pos	Neg	Neg	Pos
**2b**	93	13.74	High	Yes	4.68	Soluble (m)	High	54.8	No	Medium	NM	Neg	Neg	Neg	Neg
**3a**	92	14.56	High	Yes	4.93	Soluble (m)	High	49.2	No	Medium	M	Neg	Neg	Neg	Pos
**3b**	94	14.87	High	Yes	5.19	Soluble (m)	High	52.9	No	Medium	NM	Neg	Neg	Neg	Neg
**4a**	92	12.24	High	Yes	4.19	Soluble (m)	High	49.9	Yes	Medium	M	Pos	Neg	Neg	Neg
**4b**	95	12.74	High	Yes	4.45	Soluble (m)	High	53.4	No	Medium	M	Neg	Neg	Pos	Neg
**5a**	93	12.79	High	Yes	4.49	Soluble (m)	High	43.6	Yes	Medium	M	Neg	Neg	Pos	Pos
**5b**	95	13.24	High	Yes	4.75	Soluble (m)	High	50.1	No	Medium	M	Neg	Neg	Pos	Neg

^a^ Data predicted from the PreADMET tool; ^b^ Data predicted from the SwissADME tool. Pr. = prediction; (m) = moderate; sol = solubility; GI abs = gastrointestinal absorption; sub = substrate. Ames Test: M = mutagen; NM = non-mutagen; S9 activation or non-activation = metabolic activation.

## Data Availability

Not applicable.
